# Establishing and maintaining the data-base required for cost analysis in a clinical laboratory

**DOI:** 10.1155/S1463924683000218

**Published:** 1983

**Authors:** George E. Westlake

**Affiliations:** George Westlake Associates 232 Evergreen Drive Kentfield California 94904 USA


Journal of Automatic Chemistry, Volume 5, Number 2 (April–June 1983), pages 75–78

### Establishing and maintaining the data-base required for cost analysis in a clinical laboratory

**George E. Westlake**

*George Westlake Associates, 232 Evergreen Drive, Kentfield, California 94904, USA*

### The problem

Obtaining and understanding the costs of clinical laboratory testing—as with many health services—presents a perplexing but critical task. In most laboratories the only 'cost data' available are those accumulated for routine expense accounting, but these data are usually inadequate for analysing laboratory costs. For costing purposes, common major deficiencies in accounting data are: (1) supplies are recorded at the time they are purchased rather than when they are consumed; (2) costs are assigned on the basis of supervisory responsibility or functional area, but not both; (3) goods are recorded by vendor rather than by item; (4) insufficient statistical parameters are collected; and (5) cost allocations replace direct costs. Each of these contributes to the difficulty in understanding costs and relating them to other variables, such as testing volume and level of service.

### Scope of the paper

This paper will discuss an approach to organizing cost data that helps recognize and avoid these problems by building on simple relationships. It has been called 'inspired common sense' or, in technical jargon, 'normalizing relational data sets'. When designing a data-base and defining the data elements to be included, attempting to 'normalize' the data sets will quickly identify which questions can or cannot be answered explicitly by the available cost measurements, and which require estimates based on incomplete or complex data.

It is helpful to remember that cost data consist of two components: statistical and financial. These data become useful information only when they are relevant to the questions being asked. Questions involving cost range from economics (how does the cost of laboratory testing compare to the medical benefits?), to cost reimbursement (what portion of total operational costs should be paid for a specific group of services?), to operational policy (how can cost be reduced in my laboratory?).

The approach described will deal only with costs as they relate to operational policy.

Typical questions concerning operational laboratory costs would be:

(1) How much will it cost to increase testing volume by 10%? (How will cost change with volume?)
(2) How did costs change when the method was changed? (How did cost change when the factors of production changed?)
(3) Did changing vendors reduce cost? (How did cost change with a different mix of prices?)

The challenge: which statistical and financial data elements will help answer these questions, and how should these data be organized?

### What is a data-base?

When used for cost analysis, a data-base is a collection of interrelated financial and statistical time-series data which are used for making comparisons and projecting trends.

Anyone attempting to determine laboratory costs will soon discover that simply having access to those data that have been accumulated for general accounting will not provide satisfactory answers to most cost-related problems. A data-base organized to answer one type of problem may be useless for other types, even if many of the same data are involved. Data will be of greatest general use if aggregated data are broken down to simple data elements, all relevant data elements are present, and data are stored in functionally related groups.

An analogy would be building a house with prefabricated units, versus starting with boards and nails. The prefabricated forms allow only preconceived final results. Starting with basic building materials, although requiring more effort and planning, allows the creation of many types of structures.

The approach to be described offers rules for disassembling data structures to their basic elements. It is termed 'normalizing a relational data-base'. Depending on its compliance with certain criteria, the data-base is classified as first normal form, second normal form, or third normal form. Since these terms are part of a specialized data-base vocabulary and may not be familiar to the reader, an explanation is in order.

### *A short explanation of relational data-base terminology*

A relational data-base can be viewed as a collection of lists, each kept on a separate sheet of paper. Each list is termed a file or relation and has a title or relation name. Under the title is a series of columns, each with a label. These are the data elements (sometimes called domains or fields). Each line or row is a record (sometimes called tuple) which is composed of the data elements. A record is identified by one (or more) data element(s). This identifying tag is called the primary key. Each key must be unique so that the correct record can be retrieved and processed. Therefore, for each record in a relation (file), the data elements are the same although their values may vary.

*Example: normalizing a mailing list*

Relation name: Mailing list
Primary key: NAME

Column headings (Data elements)

| | Name | Occupation | Street | City | State |
|---|---|---|---|---|---|
| Record#1 | Jones | Lawyer | Main St. | Boston | Mass. |
| Record#2 | Smith | Banker | Center Ave. | Springfield | Il. |
| Record#3 | Foster | Chemist | Arbor Dr. | Yakima | Wash. |

*First normal form:* The criterion for first normal form is that the relation can have no repeating elements. An example of a repeating element would be trying to keep track of both a business address and a home address for each NAME. The primary key, NAME, would not be unique for the record since both the business address and home address would have the same key. In order to normalize the relation, we could add another data element, TYPE-OF-ADDRESS and make a new primary key of NAME+TYPE-OF-ADDRESS. Each record will now have a unique primary key. An alternative would be to have a separate list or relation for each NAME which would have all associated addresses. If you can record data in the above form, then it has no repeating elements, is normalized (in first normal form), and can be stored in a relational data-base.

*Second normal form:* The criterion for second normal form is that each non-key element be fully dependent on the entire primary key. This ensures that each record constitutes a discrete entity. In the Mailing List relation, when TYPE-OF-ADDRESS is added to the relation it is necessary to change the primary key to NAME+TYPE-OF-ADDRESS in order to specify which address is desired for a specific individual. Is this relation now in second normal form? No, since OCCUPATION is not dependent on the entire key. Only the name is required in order to specify OCCUPATION. The new key for the relation designates it as an entity containing address information. OCCUPATION is not directly related to TYPE-OF-ADDRESS and should be moved to another relation. From the standpoint of being able to recall data elements, we now have a satisfactory second normal form. However, the rules for normalizing give no guarantee that all relevant data elements have been included. For cost analysis, second normal form indicates that all costs in the relation are directly related to the primary key and, therefore, are traceable to that entity.

*Third normal form:* The criterion for third normal form is that the non-key data elements be entirely independent of each other. If a dependence exists, then there is a relationship between data elements that cannot be entirely defined by the primary key, or there are redundant data. For example, is there a dependence between CITY and STATE that should be removed? That is, knowing the CITY can the STATE be defined? Since several cities with the same name may exist in several states, we can conclude that no dependence exists. The Mailing List relation is in third normal form. In cost-accounting terms, third normal form means that a record consists of fundamental costs and statistics that are directly traceable to a specific entity (the primary key).

Relationships or functional links between relations are based on similar data elements. The Mailing List could be linked by NAME to an Orders Received relation to create a report describing the orders placed by those on the mailing list or listing new customers who should be added to the list.

*Table 1. Cost-related data elements.*

| |
|---|
| Relation name: Procedure cost |
| Primary key: TEST-NAME |
| Data element name: TEST-VOLUME |
| Data element name: NUMBER-OF-CAP-WORK-LOAD-UNITS |
| Data element name: LABOR-EXPENSE |
| Data element name: CONSUMABLE-EXPENSE |
| Data element name: INSTRUMENT-EXPENSE |

This approach can be applied to laboratory costs. Examine the data elements in table 1. These have been selected as important when comparing the cost of one analytical procedure to an alternative.

### *Normalizing the relation*

### *First normal form*

The criterion for first normal form is that the relation can have no repeating elements. Attempting to arrange the data elements in a two-dimensional table, with TEST-NAME as the primary key, reveals the first problem. Several of the data elements, such as TEST-VOLUME, are recorded periodically and are repeating elements. A relation called Test Volume containing the data elements TEST-NAME, DATE, VOLUME, and NUMBER-OF-CAP-WORK-LOAD-UNITS will partially resolve this problem. As an aside, the term 'volume' is ambiguous since it can refer to the number of patient analyses, or the total number of analytical cycles (including standards, controls, and repeats). Both should be included since they represent different but equally useful measures of output.

CONSUMABLE-EXPENSE has within it two repeating groups since several types of consumables are used for a given test and the price for each consumable may change with time. Establishing one relation called Method File with the elements METHOD-NAME, CONSUMABLE-NAME, and AMOUNT-PER-CYCLE and another relation called Consumables with the data elements CONSUMABLE-NAME, DATE, QUANTITY-USED, PRICE, and VENDOR normalizes these data.

Additional repeating elements are associated with INSTRUMENT-EXPENSE. Several tests may be performed on one instrument, and this mix may vary with time. Indeed, the instrument may be moved from one work-station to another. Only two data elements are directly associated with the instrument and date but not with the mix of tests performed. These are LEASE-COST (or depreciation) and MAINTENANCE-COST. To link the INSTRUMENT-COST to the operational pattern at any given time, a relation called Test Operation Profile is established which has the key TEST-NAME+DATE and the non-key elements METHOD-NAME, INSTRUMENT, and WORK-STATION.

Arranging the data in first normal form has broken complex relationships out into simpler subsets, each record of which can be accessed with a unique primary key. These are now examined for compliance with second normal form.

### *Second normal form*

The criterion for second normal form is that each non-key element be fully dependent on the entire primary key. To meet this criterion, the value of each non-key element must relate to the value of the primary key. Conversely, the primary key must be able to uniquely specify the values of each of the non-key elements. If the primary key is compound, then the non-key values must vary with (be dependent on) each of the elements of the primary key.

LABOR-EXPENSE presents a good example since it is one of the most complex issues. Simply knowing the TEST-NAME or even TEST-NAME+TEST-VOLUME will not uniquely specify LABOR-EXPENSE. Recall that in many cases several tests are performed at one work-station, which is staffed by multiple technologists each paid at a different rate. This complexity is recognized by dividing LABOR-EXPENSE into two separate relations: Staffing and Salary History. Staffing has the primary key EMPLOYEE NAME+DATE and the non-key elements WORK-STATION and HOURS-WORKED. Salary History also has a primary key of EMPLOYEE-NAME+DATE but has the non-key element of SALARY.

Another relation which may not be in second normal form is the Method File.

Primary key: METHOD-NAME + CONSUMABLE-NAME
Non-key: AMOUNT-PER-CYCLE

METHOD-NAME refers to a general chemical method which can be performed with several types of equipment with resulting variation in AMOUNT-PER-CYCLE. To take this into account, the INSTRUMENT data element must be added to the relation and concatenated with the primary key. This also accounts for simultaneously having multiple methods for one test each using a different INSTRUMENT.

*Third normal form*

The criterion for third normal form is that the non-key elements be entirely independent of each other. A data element of the Test Volume relation violates the rules of third normal form. This element is CAP-WORKLOAD-UNITS. These units are computed by multiplying the number of analyses performed by a CAP work-load factor. Thus, they are related to TEST-VOLUME (another non-key element) and they are not wholly dependent on the key (TEST-NAME) but depend, in addition, on the actions of the College of American Pathologists' work-load committee and their assignment of values. Since they can be calculated from the volume for each test, plus a table relating test type to work-load factor, CAP units should be set up as a separate relation which includes TEST-NAME, DATE, INSTRUMENT, and WORK-LOAD-UNIT. Table 2 shows the relations in third normal form.

*Table 2. Simplified relations.*

Relation name: Test Volume
Primary key: TEST-NAME + DATE
Non-key data elements: ANALYTICAL-CYCLES
NUMBER-OF-PATIENT-ANALYSES

Relation name: CAP-Work-load
Primary key: TEST-NAME + INSTRUMENT + DATE
Non-key data elements: WORK-LOAD-UNIT

Relation name: Test Operation File
Primary key: TEST-NAME + DATE
Non-key data elements: METHOD-NAME
INSTRUMENT

Relation name: Method File
Primary key: METHOD-NAME + CONSUMABLE-NAME + INSTRUMENT
Non-key data elements: AMOUNT-PER-CYCLE

Relation name: Instrument Cost
Primary key: INSTRUMENT-NAME + DATE
Non-key data elements: LEASE-COST
MAINTENANCE-COST

Relation name: Consumables Cost
Primary key: CONSUMABLE-NAME + DATE
Non-key data elements: QUANTITY-USED
PRICE
VENDOR

Relation name: Staffing
Primary key: EMPLOYEE-NAME + DATE
Non-key data elements: WORK-STATION
HOURS-WORKED

Relation name: SALARY-HISTORY
Primary key: EMPLOYEE-NAME + DATE
Non-key data elements: SALARY

### Linking simple relations to form new relations

Functional links between relations are provided by common data elements. Thus a complex question, such as 'How will costs change if the method is changed', can be answered by a three-step process:

(1) Identifying the relevant cost elements (those costs that will change depending on the decision taken).
(2) Making assumptions for future conditions such as volume increase or change in salary.
(3) Linking the required relations through common data elements to calculate actual costs without change and estimated costs with change.

*Example*

Project the change in cost that would occur with a change from method A to method B. Method B will use the same instrument and work-station as method A. A new more stable reagent will be used which costs 10% more than the current reagent and uses the same volume per instrument cycle. The CAP work-load units are the same for both procedures.

(1) Identify the relevant costs. In this example, many costs will remain the same. The question is, will the increased stability and shelf-life decrease wastage and more than make up for the higher price?
(2) Make assumptions for future conditions. Assume that no change in test volume will occur.
(3) Link the required relations. In this case, the figure desired, current reagent wastage, was not explicitly addressed in the original data-base design. To form this new relation, link the TEST-NAME to the Test Procedure file. This identifies the METHOD-NAME for the past DATE. METHOD-NAME plus CONSUMABLE-NAME and INSTRUMENT accesses the AMOUNT-PER-CYCLE.

TEST-NAME plus DATE will yield ANALYTICAL-CYCLES for the DATE. From the values for AMOUNT-PER-CYCLE and ANALYTICAL-CYCLES, the quantity that would have been consumed without wastage can be computed. Using the CONSUMABLE-NAME and DATE as the key in the Consumables relation will yield actual volume and cost. (Assume that the period being examined is long enough to average out inconsistencies in purchase versus usage.) The difference in computed usage and actual can be multiplied by the unit PRICE for the DATE to get the cost of wastage with the current reagent.

Since all other factors are constant, the answer to the question concerning potential savings due to decreased reagent wastage will hinge on whether there is a high probability that the more stable reagent will cut current wastage by more than 10%. If the data from our data-base show high waste, then switching reagents might reduce cost. If wastage is currently less than 10%, then no change should be made on the basis of cost.

The ability to answer questions concerning relationships that were not specifically addressed in the original design is one of the benefits of relational data-bases in third normal form. Simple relationships composed of fundamental costs can be combined to form new complex relations.

Conversely, if the data-base cannot answer a question, the assumption can be made that such data are not available without making simplifying assumptions. This will occur in those areas in which there are joint costs of production (costs that cannot be directly traced to the entity in question). An example of a joint cost is instrument LEASE-COST and MAINTENANCE-COST for an instrument performing multiple methods.

In many cases, labour cost will be a joint cost which is traceable to a work-station but not to an individual procedure.

### Implementing the data-base

Policies and procedures established by management, and the expense of establishing and maintaining the data-base, determine which complex costs can be resolved to third normal form. If labour policy were to reimburse on a piece-work basis, then labour cost would be related entirely to test volume and pay scale. For those tests performed by outside laboratories on a fee-for-service basis, the analytical cost relates only to volume and price. Similarly, the problem of matching the consumables with the corresponding test and volume elements will be simplified if these supplies are drawn as needed from a store-room account. Thus, the usefulness of the data-base will depend not only on structure, but also on the management policies that govern the relations of the data elements.

### Summary

The advantages of implementing a normalized relational data-base are:

(1) It has a simple and easily grasped two-dimensional form.
(2) Specifying the key and non-key elements in third normal form breaks complex data into fundamental data elements and groups them in simple one-to-one relations.
(3) These simple relations can be combined to derive any complex traceable cost.

In the process, meaningful and often controllable relationships between data elements are revealed. Other operational advantages of a properly designed relational data-base are economy of storage space, accuracy, and ease of updating files.

Establishing an effective data-base for cost analysis requires knowing the relationships of those data elements that are controllable by management. Focusing on traceable costs emphasizes the entities that directly incur or determine costs and thus are to be controlled.

Although the laboratory organization and product are complex, and many cost-related questions will always require utilization of cost data which bear a complex relationship to the problem being studied, improvements can be made in current practices. For certain critical elements, changes in accounting and management policies of the laboratory should be considered in order to simplify the available data. As automation of management information systems becomes more widespread, and data-bases are better designed, there is promise of rapid access to greatly expanded relevant and explicit information with which to analyse and control clinical laboratory costs.

### Definitions

*Data element:* the smallest piece of data that is meaningful for the purpose at hand.
*First normal form:* a relation with no repeating elements.
*Joint cost:* a cost shared by two or more entities. For example, the cost of an analytical instrument would be a joint cost to test results produced.
*Normalized* (first normal form): a two-dimensional file without repeating data elements.
*Primary key:* the data element (or group of data elements) which uniquely identifies the record. Data elements not included in the primary key are termed 'non-key' elements.
*Record:* a row in a relational data-base.
*Relation:* a file represented in a normalized two-dimensional form.
*Repeating element:* a data element which can take on more than one value in its relation to the record's primary key.
*Second normal form:* a normalized relation in which all of the non-key elements are functionally dependent on the primary key.
*Third normal form:* a relation which is in second normal form and in which the non-key elements are mutually independent.
*Traceable cost:* a cost that can be associated with a specific entity.

### Bibliography

Codd, E. F., *Communications of the ACM* (June 1970).
Gane, C. and Sarson, T., *Structured Systems Analysis: Tools and Techniques* (Prentice-Hall, Inc., Englewood Cliffs, New Jersey, 1979).
Martin, J., *Computer Data-Base Organization* (Prentice-Hall Inc., Englewood Cliffs, New Jersey, 1975).

### SYMPOSIUM

*International Symposium on Chromatography and Mass Spectrometry in Nutrition Science and Food Safety*

The meeting, which is to be held at the Hotel Eden au Lac in Montreux, Switzerland, from 20 to 22 June 1983, is being arranged by the Italian Group for Mass Spectrometry in Biochemistry and Medicine together with Nestlé Products Technical Assistance Company Ltd. All the latest aspects of chromatography and mass spectrometry in nutrition science and food safety are intended to be illustrated and discussed. Main topics are: food science, flavours and aromas, nutritional biochemistry in humans and animals, disease in relation to nutrition, food safety, and improvements in the methodology of chromatography and mass spectrometry in nutrition science and food safety. The Chairmen are Drs Alberto Frigerio (Italian Group for Mass Spectrometry in Biochemistry and Medicine) and Hubert Milon (Nestlé Products Technical Assistance Company Ltd).

*Further information from the Italian Group for Mass Spectrometry in Biochemistry and Medicine, Via Eritrea 62, I 20157 Milan, Italy.*

